# Wound Irrigation Using Wet Gauze May Reduce Surgical Site Infection Following Laparoscopic Appendectomy

**DOI:** 10.3389/fsurg.2022.813738

**Published:** 2022-02-08

**Authors:** Abdullah Al-Sawat, Ji Yeon Mun, Sung Hoon Yoon, Chul Seung Lee

**Affiliations:** ^1^Department of Surgery, College of Medicine, Taif University, Taif, Saudi Arabia; ^2^Department of Surgery, St. Vincent's Hospital, College of Medicine, The Catholic University of Korea, Suwon, South Korea; ^3^Department of Surgery, Seoul St. Mary's Hospital, College of Medicine, The Catholic University of Korea, Suwon, South Korea

**Keywords:** surgical wound infection, appendectomy, laparoscopy, wound irrigation, length of stay

## Abstract

**Purpose:**

This study aimed to compare the perioperative outcomes of wet gauze and conventional irrigation after laparoscopic appendectomy to determine whether wet gauze irrigation can help reduce surgical site infection (SSI).

**Methods:**

A total of 308 patients undergoing laparoscopic appendectomy were included in this study between December 2018 and May 2020. Of these, 132 (42.9%) received gauze irrigation (group 1), and 176 patients (57.1%) received conventional irrigation (group 2). Pre-operative outcomes and complications, including SSI, were compared after propensity score matching (PSM) to adjust for baseline differences and selection bias.

**Results:**

After 1:1 PSM, 92 well-matched patients in each group were evaluated. Regarding perioperative outcomes between groups 1 and 2, the rate of severe complications (Clavien-Dindo Classification grades III, IV, and V), operative time, and readmission rate did not differ between the groups. Superficial/deep SSIs were observed more frequently in group 2 (8/92 cases) than in group 1 (1/92 cases; *p* = 0.017). The organ/space SSIs rate was not significantly different between the two groups (1/92 group 1 and 0/92 group 2, *p* = 0.316). However, post-operative hospital stay was significantly longer in group 2 (2.8 ± 1.3 days) than in group 1 (1.6 ± 1.2 days; *p* < 0.001). In the univariate analyses, wound irrigation using wet gauze was an independent protective factor for superficial or deep SSI (*p* = 0.044).

**Conclusions:**

Wound irrigation using wet gauze after fascia closure has a significant beneficial effect on reducing post-operative superficial/deep SSI following laparoscopic appendectomy.

## Introduction

Acute appendicitis is one of the most common causes of emergency abdominal surgery worldwide (1). The management of acute appendicitis has changed over the last few decades. The open approach, which involves a single surgical incision through McBurney point, is the standard of care (2). When compared to minimally invasive techniques, the open approach has the advantages of shorter operative times and less intra-abdominal abscess formation (3). On the other hand, the open technique is associated with a greater likelihood of wound infection, unfavorable cosmetic outcomes, and a longer post-operative stay (4).

Recently, the incidence of laparoscopic appendectomy (LA) using single-incision laparoscopic appendectomy (SILA) or conventional laparoscopic appendectomy (CLA) has been increasing (5). Reports showed a marked decrease in the incidence of wound infection from 8.7% with the open approach to <4.2% with the laparoscopic approach (6). Furthermore, it has advantages in terms of cosmetic outcomes and length of hospital stay (7).

Surgical site infection (SSI) is still one of the major complications after appendectomy (8). The cause is multifactorial; however, the severity of the inflammation or perforation is a key contributor (9). Thus, many adjuncts to LA have been tried, including specimen retrieval bags, wound protectors, laparoscopic abdominal irrigation, and wound irrigation using saline, povidone-iodine (PI), and antibiotics to minimize the risk of SSI after abdominal procedures (10).

As conventional wound irrigation can flush away inflammatory cells, which are vital for host defense and wound healing (11), we hypothesized that irrigation of the surgical wound using wet gauze could dilute and thus eliminate the infectious agent and reduce the risk of SSI. Therefore, this study aimed to evaluate the role of wound irrigation using wet gauze in reducing the SSI rate after LA.

## Methods and Materials

### Patients

The present study was conducted retrospectively. Patients between 15 and 75 years of age who underwent LA between December 2018 and March 2020 at Seoul St. Mary's hospital, Suwon St. Vincent's hospital, College of Medicine, The Catholic University of Korea were included. Patients who displayed factors that might affect wound healing [older than 75 years, immunocompromising disease, steroid use, American Society of Anesthesiologists (ASA) grade IV, hematologic disorder, previous abdominal operative history, body mass index (BMI) > 30], patients with appendiceal abscess (>4 cm) requiring drainage, phlegmon, or underwent open appendectomy were excluded from this study.

Pre-operative contrast-enhanced abdomino-pelvic computed tomography was used to confirm the diagnosis for all patients. An inflamed but grossly intact, non-gangrenous, non-suppurative appendix with no associated abscess or peritonitis was defined as uncomplicated appendicitis. CT features such as abscess, extraluminal air, intra- and extraluminal appendicolith, and periappendicular fluid to be defined as complicated acute appendicitis.

All patients were given a single intravenous dosage of second-generation cephalosporine 0–60 min prior to the incision. In case of complicated appendicitis, concurrent intravenous metronidazole (50 mg/kg to a maximum dosage of 2 g/day) is administered. Based on the operative findings and the patient's clinical condition, the type and length of antibiotics given are not longer than 3–5 days post-operatively (12).

**Figure 1:**
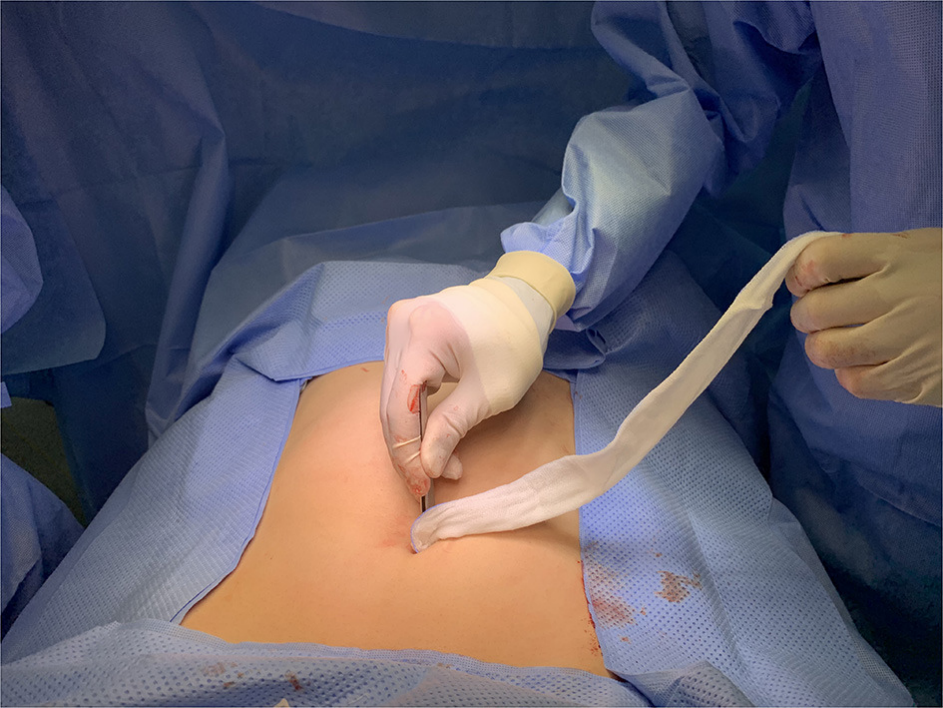
Wound irrigation using wet gauze after closure of the fascia layer. After gentle wound irrigation with wet gauze, dry gauze was used to dry the wound.

Patients were divided into two groups. Group 1 received irrigation using wet gauze with normal saline after closure of the fascia layer (Figure 1), and group 2 received conventional irrigation without gauze.

SSIs were defined according to the criteria of the National Nosocomial Infection Surveillance System (NNIS) by the Centers for Disease Control and Prevention (CDC) (13), which can be superficial, deep, and organ/space SSI. Superficial incisional SSI involves only the skin and subcutaneous tissue. Deep incisional SSI involves deep tissues, such as fascial and muscle layers, and organ/space SSI involves any part of the organs' anatomy and spaces that are incised, which were opened or manipulated during operation (14).

Our study was approved by the Institutional Review Board of the Catholic University of Korea (KC20RASIU0433).

### Irrigation Technique

Wet gauze irrigation was performed using forceps and gauze soaked in saline solution. The surgeon wiped the umbilical port site wound in concentric circles, starting directly over the closed fascia and moving outward (Figure 1). The wound is then dried using dry gauze. In contrast, conventional irrigation is performed using a piston syringe filled with normal saline at the upper edge of the umbilical port site wound. The surgeon starts to irrigate steadily and continuously without force in one direction until it is empty. Either technique was used on the umbilical port site wound for all patients.

### Surgical Procedure

The operation was performed using 3-ports or single-port LA according to the surgeon's experience. Conventional (3-port) LA was performed under general anesthesia, and euvolemia maintenance, body temperature optimization, and blood glucose control were performed during the operation. The operator's preference determined the trocar insertion site; a 10 mm trocar was introduced at the umbilicus. After achieving the pneumoperitoneum state, the other two 5 mm trocars were introduced at the left lower quadrant (LLQ) and suprapubic or LLQ and right lower quadrant sites. Appendiceal artery ligation and appendiceal base-tie by laparoscopic endoloop were performed. The appendix was retrieved through a laparoscopic bag in all cases of LA. Intra-abdominal irrigation was performed if there was an abscess or dirty fluid collection at the periappendiceal or pelvic cavity. Wound closure was performed layer-by-layer; the fascia was closed using a 1-0 or 2-0 antibiotic-coated Vicryl, and then either wet gauze or conventional irrigation of the wound was performed according to the surgeon's preference. Finally, the skin was closed using a subcuticular suture.

SILA was performed according to a previously described maneuver (15). Briefly, using the open method, a 1.5–2 cm vertical skin incision over the umbilicus was made. A glove port (431AT-2W, Nelis, Bucheon, South Korea) with three trocar channels was then inserted into the peritoneal cavity; the instruments and procedures for dissection and ligation of the appendix were the same as in the CLA. The subsequent procedure was performed in the same way as for CLA.

### Statistical Analyses

Continuous variables were expressed as mean ± standard deviation, and categorical variables were expressed as numbers (%). Differences between groups were evaluated using a Student's *t*-test and *x*^2^ test for continuous and categorical variables, respectively. To reduce selection bias due to the retrospective design of the current study, PSM was conducted. To calculate the scores of the individuals, a non-parsimonious logistic regression model was used in accordance with the pre-defined covariates, including sex, age, ASA category, BMI, and diagnosis (complicated appendicitis). The patients who underwent LA were matched based on scores from the algorithm of the nearest neighbor and 1:1 matching without specific caliper width or replacement. Statistical analysis was performed using SPSS (version 24.0; IBM SPSS Statistics^®^, Armonk, NY, USA). Statistical significance was set at *P* < 0.05.

## Results

A total of 308 patients met our inclusion criteria: 132 patients in the wet gauze wound irrigation group (group 1) and 176 patients in the conventional wound irrigation group (group 2). As shown in Table 1, there were no differences in patient characteristics between the two groups, including patient-related factors (age, BMI, ASA physical status, and underlying disease) and systemic inflammation factors (white blood cell count). The mean age of patients in group 1 and group 2 was 40.2 ± 18.8 years and 41.2 ± 18.7 years, respectively (*p* = 0.683). The mean BMI in group 1 and group 2 was 23.3 ± 3.7 kg/m^2^ and 23.3 ± 3.7 kg/m^2^, respectively (*p* = 1.000). The rate of complicated appendicitis also did not differ between the two groups (*p* = 1.000). Furthermore, no difference was found in the operative technique between the groups (*p* = 1.000).

**Table 1:** Patient characteristics.

		**Group 1 (*n* = 92)**	**Group 2 (*n* = 92)**	***P*-value**
Sex (M/F)		50/42	53/39	0.656
Age (year)		40.2 ± 18.8	41.2 ± 18.7	0.683
ASA^a^ (%)				1.000
	I	68	68	
	II	21	21	
	III	3	3	
BMI^b^ (kg/m^2^)		23.2 ± 4.4	22.9 ± 3.8	0.575
Complicated appendicitis^c^ (%)		39 (53.4)	34 (46.6)	0.932
Pre-operative white blood cell (/μL)		11846.3 ± 4253.8	12069.2 ± 4039.1	0.717
Operation type (SILA^d^ VS CLA^e^)		45/47	45/47	1.000

a *American Society of Anesthesiologists score*.

b *Body mass index*.

c *Perforated appendicitis, Gangrenous appendicitis and Peri appendiceal abscess*.

d *Single-incision laparoscopic appendectomy*.

e *Conventional three-port laparoscopic appendectomy*.

Regarding perioperative outcomes between groups 1 and 2 (Table 2), the rate of severe complications (Clavien-Dindo Classification grades III, IV, and V) and operative time were not significantly different between the groups. Superficial/deep SSIs were observed more frequently in group 2 (8/92 cases) than in group 1 (1/92 cases; *p* = 0.017). The organ/space SSIs rate was not significantly different between the two groups (1/92 group 1 and 0/92 group 2, *p* = 0.316). Post-operative hospital stay was significantly longer in group 2 (2.8 ± 1.3 days) than in group 1 (1.6 ± 1.2 days; *p* < 0.001). In contrast, readmission within 30 days showed a greater tendency in group 2, without clinical significance (*p* = 0.157). No mortality was recorded within 30 days post-operatively in either group. In the univariate analyses, wound irrigation using wet gauze was an independent protective factor for superficial or deep SSI (*p* = 0.044; Table 3). Nevertheless, patient characteristics, operative time, and techniques were not risk factors for superficial or deep SSI.

**Table 2:** Perioperative outcomes.

	**Group 1 (*n* = 92)**	**Group 2 (*n* = 92)**	***P*-value**
Operative time (>120 min)	2 (1.1)	4 (2.2)	0.406
Post-operative hospital stay (days)	1.6 ± 1.2	2.8 ± 1.3	<0.001
Total SSI^a^	2 (2.2)	8 (8.7)	0.087
Superficial/deep SSI	1 (1.1)	8 (8.7)	0.017
Organ space SSI	1 (1.1)	0	0.316
Ileus	0	0	–
Severe complications^b^ (%)	0	0	–
Re-operation (%)	0	–	–
Re-admission within 30 days (%)	0	2 (2.2)^c^	0.157
Mortality within 30 days (%)	0	0	–

a *SSI, surgical site infection*.

b *Clavien-Dindo classification ≥IIIa*.

c *1 patient: Organ space SSI, 1 patient: Abdominal pain*.

**Table 3:** Predictors of superficial/deep SSI after laparoscopic appendectomy identified using univariable logistic regression analysis.

**Variable**	**Univariate analysis OR (95% CI^a^)**	***P*-value**
Age (>55)	0.39 [0.48–3.257]	0.389
Gender (female)	2.67 [0.65–11.01]	0.175
BMI (>25 kg/m^2^)	0.94 [0.21–5.34]	0.938
ASA^b^	0.73 [0.17–3.16]	0.514
Operative time (>120 min)	0.43 [0.53–3.44]	0.424
Complicated appendicitis on initial CT	0.43 [0.09–2.20]	0.313
Pre-operative white blood cell (>12,140/μL)^c^	1.29 [0.34–4.98]	0.708
Wound irrigation using wet gauze	0.12 [0.01–0.94]	0.044
Operation type (SILA^d^ vs CLA^e^)	0.76 [0.20–2.91]	0.684

a *CI, confidence interval*.

b *American Society of Anesthesiologists score*.

c *Median value*.

d *Single-incision laparoscopic appendectomy*.

e *Conventional three-port laparoscopic appendectomy*.

## Discussion

According to our findings, wound irrigation using wet gauze was an independent protective factor for superficial/deep SSI development after LA compared to conventional saline irrigation. Moreover, the group that received wound irrigation using wet gauze experienced a significantly shorter length of hospital stay.

Wound infection and intra-abdominal abscess are considered the most typical post-operative complications after appendectomy (16). Extensive attempts have been made to reduce the rate of such complications. For example, some studies advocate using a retrieval bag to prevent direct contact of the inflamed appendix with the incision to decrease the SSI rate (17). Others support intra-abdominal lavage to reduce the risk of intra-abdominal abscess formation, which further decreases wound infection rate (15). Lee et al. (18) suggested reducing the operative time and disinfecting the umbilicus to reduce SSIs.

In addition to the previously described methods, post-operative application of a wide range of disinfectants, such as wound saline irrigation, PI, chlorhexidine gluconate irrigation, and antibiotic powder, has been demonstrated to reduce the SSI rate (19). Furthermore, eliminating the debris, tissue exudates, and bacterial load of the surgical incision using post-operative wound irrigation has been demonstrated to improve SSI rates by creating an optimal environment for wound healing (20). Unfortunately, wound irrigation following LA has seldom been reported. Therefore, a double-blind randomized trial on patients with acute appendicitis was conducted for 205 patients who underwent open appendectomy and were divided into three groups: no wound irrigation, saline irrigation, or antibiotic-saline irrigation (21). They found wound irrigation significantly reduced incisional SSI rates. Adding antibiotics to a saline solution did not affect the outcome or decrease SSI.

Many studies have been conducted on wound irrigation during different types of surgery. For example, Onishi et al. (22) showed that using saline wound irrigation and PI intraoperatively was associated with a reduced incidence of SSI following spinal surgery. Another study showed that using chlorhexidine gluconate wound irrigation when antibiotics were not given within 60 min pre-operatively was associated with a reduced incidence of SSI following cesarean section (23). Therefore, regardless of the procedure, we believe that wound irrigation using wet gauze is a safe and straightforward practice that could improve the surgical outcome and be used post-operatively in clinical practice.

Surgeons tend to use less invasive techniques when managing acute appendicitis. Although single-incision LA is more attractive due to less abdominal wall trauma, faster recovery (24), and better cosmetic results compared to CLA (15). Moreover, Markar et al. (25) found in their meta-analysis that the incidence of wound infection was reported in all the included studies, with 4% in each group and no statistical difference between the two techniques. Interestingly, Lee et al. (18) studied more than 2,500 patients who underwent either SILA or CLA and found that superficial incisional SSI was significantly higher in the SILA group, which can be explained by the longer operative time and excessive traction on the incision causing a delay in the wound healing due to hypoxia. In contrast, our study showed that SILA is not inferior to CLA with regard to the SSI rate.

Procedure-related factors are not the only reasons for SSI. Patient-related factors, for example, advanced age and underlying disease, play a significant role in wound healing and may increase SSI risk (26). However, we did not find a significant effect of patient-related factors on SSI rate in our analysis.

The current study was conducted in a multicenter setting, and exclusion criteria for risk factors of SSI were set to overcome biases. Furthermore, although PSM analysis was performed to account for bias, it is important to note that this study has limitations. Since this study had a non-randomized retrospective design with limited sample size, the decision to perform CLA or SILA was based on the surgeon's experience, and the inability to standardize the post-operative care might introduce a bias. Therefore, further prospective studies on factors associated with SSI are critical.

## Conclusions

Wound irrigation using wet gauze after fascia closure has a significant beneficial effect on reducing post-operative superficial/deep SSI following LA and decreasing the length of hospital stay. Nevertheless, wet gauze wound irrigation can be safe and effective in different surgical incisions, but a large-scale retrospective study or randomized controlled trial is required.

## Data Availability Statement

The original contributions presented in the study are included in the article/supplementary material, further inquiries can be directed to the corresponding author/s.

## Ethics Statement

This study was approved by the Institutional Review Board of the College of Medicine, the Catholic University of Korea (KC20RASIU0433). Written informed consent for participation was not required for this study in accordance with the national legislation and the institutional requirements.

## Author Contributions

AA-S and CL: conceptualization and design. JM and SY: patient data and samples. AA-S, JM, and SY: experiments, collection, assembly of data, and manuscript writing and editing. AA-S, SY, and CL: data analysis and interpretation. CL: critical revision. All authors have read and agreed to the published version of the manuscript.

## Conflict of Interest

The authors declare that the research was conducted in the absence of any commercial or financial relationships that could be construed as a potential conflict of interest.

## Publisher's Note

All claims expressed in this article are solely those of the authors and do not necessarily represent those of their affiliated organizations, or those of the publisher, the editors and the reviewers. Any product that may be evaluated in this article, or claim that may be made by its manufacturer, is not guaranteed or endorsed by the publisher.
